# Sex and race/ethnicity differences in the 24-hour movement behaviors among adolescents in the Future of Families and Child Wellbeing Study (FFCWS)

**DOI:** 10.1007/s40615-025-02796-w

**Published:** 2025-12-22

**Authors:** Tiwaloluwa A. Ajibewa, Lindsay Master, Gina Marie Mathew, David A. Reichenberger, Orfeu M. Buxton, Anne-Marie Chang, Lauren Hale

**Affiliations:** 1 Department of Preventive Medicine, Feinberg School of Medicine, Northwestern University, Chicago, IL, USA; 2 Department of Biobehavioral Health, College of Health and Human Development, Pennsylvania State University, University Park, PA, USA; 3 Program in Public Health, Department of Family, Population, and Preventive Medicine, Renaissance School of Medicine, Stony Brook University, Stony Brook, NY, USA; 4 Oregon Institute of Occupational Health Sciences, Oregon Health & Science University, Portland, OR, USA; 5 Knight Cardiovascular Institute, Oregon Health & Science University, Portland, OR, USA

**Keywords:** Movement behaviors, Race/ethnicity, Sex, Adolescence

## Abstract

**Purpose:**

To compare components of the 24-hour movement behaviors (physical activity, sedentary behavior, sleep) across sex and race/ethnic groups among a diverse sample of adolescents to identity potential gaps and opportunities for intervention.

**Methods:**

The sample consisted of 704 adolescents (15.4±0.6 years; 51% Black; 53% female) from year 15 of the Future of Families and Child Wellbeing Study. Twenty-four-hour movement behaviors were measured over a week-long period using waist- and wrist-worn accelerometry. Sex, racial, and ethnic differences in 24-hour movement behaviors were examined with chi-square and *t*-test analyses.

**Results:**

Female adolescents, on average, spent less time in light physical activity (LPA) and in moderate-to-vigorous physical activity (MVPA) than male adolescents. Additionally, female adolescents spent more time in sedentary behavior and had longer daily total sleep time than adolescent males. Black adolescents had higher average LPA than White adolescents. Black adolescents also had higher average MVPA and LPA than Hispanic/Latino adolescents. Hispanic/Latino youth had longer total sleep time, and more sedentary time than Black youth. No other significant differences were observed across these demographic groups.

**Conclusions:**

There is a continued need for interventions to promote physical activity and sleep among adolescents, with a particular focus on increasing sleep duration among boys and physical activity among girls and Hispanic/Latino adolescents.

## Introduction

Within the scientific community, there is recognition that movement behaviors—physical activity, sleep, and sedentary behavior—are interdependent and thus should not be examined in isolation [1]. This acknowledgement has led to studying movement behaviors through what is known as the 24-hour activity cycle (i.e., 24-hour movement behaviors). The 24-hour activity cycle seeks to integrate all movement activities within the 24-hour period to understand how the time spent in each movement activity interacts to influence holistic health [2]. However, differences in 24-hour movement behavior research among samples of racial and ethnically diverse adolescents remain largely underexplored. Examining these differences across movement behaviors provides a foundational basis for understanding how to tailor future interventions to adequately address the potentially different barriers or challenges affecting certain demographic groups disproportionately. Such interventions can then help guide and inform policies. Additionally, by improving movement behaviors particularly in adolescence, benefits such as improved overall health outcomes and decreased comorbidities could lower the financial and societal burden of preventable conditions [3, 4].

Adolescence is a critical developmental period wherein reductions in health-enhancing behaviors such as habitual physical activity engagement and sufficient sleep duration occur [5, 6]. Marked reductions in physical activity are particularly notable among adolescent girls and racial and ethnic minoritized youth, with a similar pattern observed for sleep behavior [6–12]. According to the life course health development framework, childhood antecedents contribute over time to health outcomes and subsequent disparities in adulthood [13]. Specifically, the life course theory posits that early life experiences, such as exposures to structurally patterned physical, environmental, and psychosocial factors, particularly during sensitive periods (e.g., adolescence), result in long-term shifts in health trajectories that may persist despite interventions later in life [14]. Therefore, adolescence is a critical stage for developing healthy behaviors, and where modifiable risk behaviors (e.g., sedentary behavior, physical inactivity) are established [15]. With emerging evidence suggesting that health behaviors such as physical activity during this period influence mental (e.g., depression) and physical (e.g., obesity) health trajectories into adulthood and beyond [16], it becomes necessary to understand and intervene during adolescence, especially among racial and ethnic minoritized youth, to help shape behavioral trajectories as they develop into adults.

Guidelines for 24-hour movement behaviors have been established for Canadians [17] and adopted by other countries and regions such as South Africa, New Zealand, and the Asia-Pacific region [18]. However, such recommendations were not adopted in the most recent United States (US) physical activity guidelines [19]. The US physical activity guidelines recommend that adolescents engage in at least 60 daily minutes of moderate-to-vigorous physical activity (MVPA), and the World Health Organization (WHO) recommends that adolescents obtain 8–10 h of sleep, and no more than 2 h of screen time, during which the individual is typically sedentary [19, 20]. Despite these recommendations, adolescents often do not meet such guidelines. For example, recent evidence from the Youth Risk Behavior Survey has indicated that less than one-quarter of U.S. teenagers (23.9%) meet the recommended 60 min of MVPA per day [21]. Similarly, investigators using the same survey to examine meeting the US sleep guidelines [22] found that more than 60% of adolescents were not meeting the sleep recommendations [23, 24]. When considering the lower levels of physical activity among minoritized youth, coupled with existing health disparities across gender and race/ethnic groups observed in sleep and sedentary behavior [7, 25, 26], there is a critical need to document potential racial, sex, and gender differences in 24-hour movement behaviors among minoritized groups of adolescents. This, in turn, will help create future behavioral health interventions that adequately address their unique needs.

The primary objective of the present study is to characterize 24-hour movement behaviors [MVPA, light physical activity (LPA), sedentary behavior, and sleep] using objective measures among a diverse sample of adolescents. Given the documented disparities in physical activity and sleep, we hypothesized that female adolescents would have lower levels of physical activity and higher levels of sedentary behavior, but longer sleep duration, compared with their male peers within the sample. Similarly, we hypothesized that non-Hispanic Black and Hispanic adolescents would have lower levels of physical activity, higher levels of sedentary behavior, and shorter sleep duration across the 24-hour period compared with their non-Hispanic White peers.

## Methods

### Participants

Data from the Future of Families and Child Wellbeing Study’s (FFCWS) sleep sub-study were used in the present analyses. The FFCWS is a national, longitudinal birth cohort study of children born between the years of 1998 and 2000 in the US [27]. The FFCWS oversampled births to unmarried mothers at baseline, and when weighted, data from the study participants are representative of births in large US cities during the years of 1998, 1999, and 2000. The cohort has been followed since birth at subsequent waves when the focal children were approximately 1, 3, 5, 9, 15, and 22 years of age. The current analyses used data from year 15 of the study when participants were approximately 15 years of age in a randomly selected sub-sample of participants who took part in the FFCWS sleep sub-study. Given the objective of this present study was to characterize 24-hour movement behaviors during the adolescent life stage, data from year 15 were the only data used. Participants who agreed to take part in the sleep sub-study (*n* = 1,049) were asked to concurrently wear an accelerometer on their non-dominant wrist and another accelerometer on their hip for seven consecutive days. Greater detail regarding the FFCWS sample and overall design can be found elsewhere [27]. The Princeton University Institutional Review Board (IRB) approved the FFCWS study protocol, with primary caregivers providing informed consent and adolescents providing assent.

### Inclusion and Exclusion Criteria

Participants were presently included if they had a minimum of 4 valid days of sleep and activity data. A total of 774 participants with complete sleep and physical activity data were initially included. However, there were much fewer adolescents who identified as Asian (*n* = 23) and Multiracial (*n* = 47) versus those who self-identified as Hispanic/Latino, non-Hispanic White, or non-Hispanic Black. As such, we excluded Asian and Multiracial youth from analyses to preserve the validity of our findings, resulting in a final sample size of 704 participants.

### Measures

#### Sociodemographic Factors

All variables were measured during the year 15 wave of the FFCWS except sex. The main exposure variables of interest were the participants’ sex assigned at birth and race/ethnicity. Sex was reported by caregivers at the youth’s birth, whereas race and ethnicity were self-reported on the FFCWS year 15 survey. Other sociodemographic variables included body mass index (BMI) for-age and sex percentile, annual household income, and primary caregiver education level, self-reported on the FFCWS survey. BMI percentile was constructed and calculated using in-home measured height and weight or teen self-report together with the 2000 Centers for Disease Control and Prevention’s (CDC) growth charts [28]. Self-reported timing of pubertal maturation was assessed via the Pubertal Development Scale using a single item that asks whether the adolescent’s physical development was earlier, about the same, or later than most [29]. Annual household income in USD and primary caregiver education level (less than high school; high school diploma/GED; some college; Bachelor’s degree or greater) were self-reported by the primary caregiver at year 15. The additional sociodemographic characteristics beyond sex and race/ethnicity were included to give contextual insight into the adolescent study sample and were not covariates in this descriptive study.

#### Physical Activity

Physical activity was assessed using the waist-worn GT3X Actigraph accelerometer (Ametris formerly ActiGraph LLC; Pensacola, FL) at a frequency of 80 Hz. All participants were asked to wear the tri-axial accelerometer for seven days snugly on their right hip using an elastic belt. The 24-hour movement behaviors were categorized using a neural network classifier [30] into minutes spent in sedentary behavior, LPA, or MVPA. Overall wear time was determined by a validated algorithm within the Actilife software [31]. Consecutive periods of zero counts for ≥ 90 min were defined as non-wear time, as well as 2-minute intervals of nonzero counts bounded by 30-minute consecutive zero counts resulting from artifactual movement. A day was determined as invalid if there was > 25% of non-wear minutes in the wake portion of the day. Non-wear times were excluded from the processed data and only valid days were used in the analysis. Meeting the physical activity recommendations was defined as engaging in at least 60 min of MVPA/day.

#### Sleep

Sleep measures were collected using a wrist-worn Actiwatch Spectrum (Philips Respironics; Murrysville, PA) accelerometer. Participants were instructed to wear the accelerometer on their non-dominant wrist for seven days for an objective measurement of sleep behavior. At least two independent scorers determined cut-point, validity of days, and set sleep intervals. Additional details on sleep scoring and valid days can be found elsewhere [32]. Total sleep time was defined as the 24-hour total sleep time including number of minutes of sleep during the nighttime sleep interval as well as daytime naps. Meeting the sleep recommendations was defined as obtaining a minimum of at least 8 h of sleep per day.

#### Alignment of Physical Activity and Sleep

Sleep and activity data for each participant were merged at the 1-minute level to accurately capture true non-sleep sedentary behavior, which excluded scored nighttime sleep and daytime nap intervals. Daily time spent in each activity category was calculated as minutes/day during the waking (non-napping) hours of each valid day, defined as the period between first waking up for the day and before falling asleep at night, as determined by sleep scoring. Daily minutes in sedentary behavior, LPA, MVPA, and total sleep time were averaged across valid days for each participant.

### Statistical Analyses

All data analyses were conducted using Stata 17.0 (StataCorp LP, College Station, TX) and SAS v9.4 (SAS Institute, Cary, NC). A series of *t*-tests were conducted to examine differences in 24-hour movement behaviors by sex and race groups. Chi-square tests were conducted to examine differences across sex and race groups between those meeting and not meeting guidelines for physical activity and sleep. Adjusted p-values were calculated for each of the four components of the 24-hour movement behaviors (total sleep time, sedentary time, LPA, and MVPA) using false discovery rate (FDR) corrections (proc multtest procedure in SAS specifying FDR). An alpha level of *p* <.05 was used for this analysis, with raw p-values presented alongside FDR adjusted p-values.

## Results

### Sample Characteristics

Participant characteristics are displayed as mean and standard deviation or percentage in Table 1. Study participants had a mean±*SD* age of 15.4±0.6 years, were 52.7% female (48.3% male), were predominantly non-Hispanic Black (51.4%), and had an average BMI-for-age and sex percentile of 71.3±25.9. Regarding 24-hour movement behaviors, participants spent on average of 387 min/day in sedentary time, 502 min/day in LPA, 29 min/day in MVPA, and 445 min/day sleeping. On average, participants were active (i.e., in LPA or MVPA) for approximately 531 min/day.

### Sex Differences in 24-Hour Movement Behaviors

**Fig. 1:**
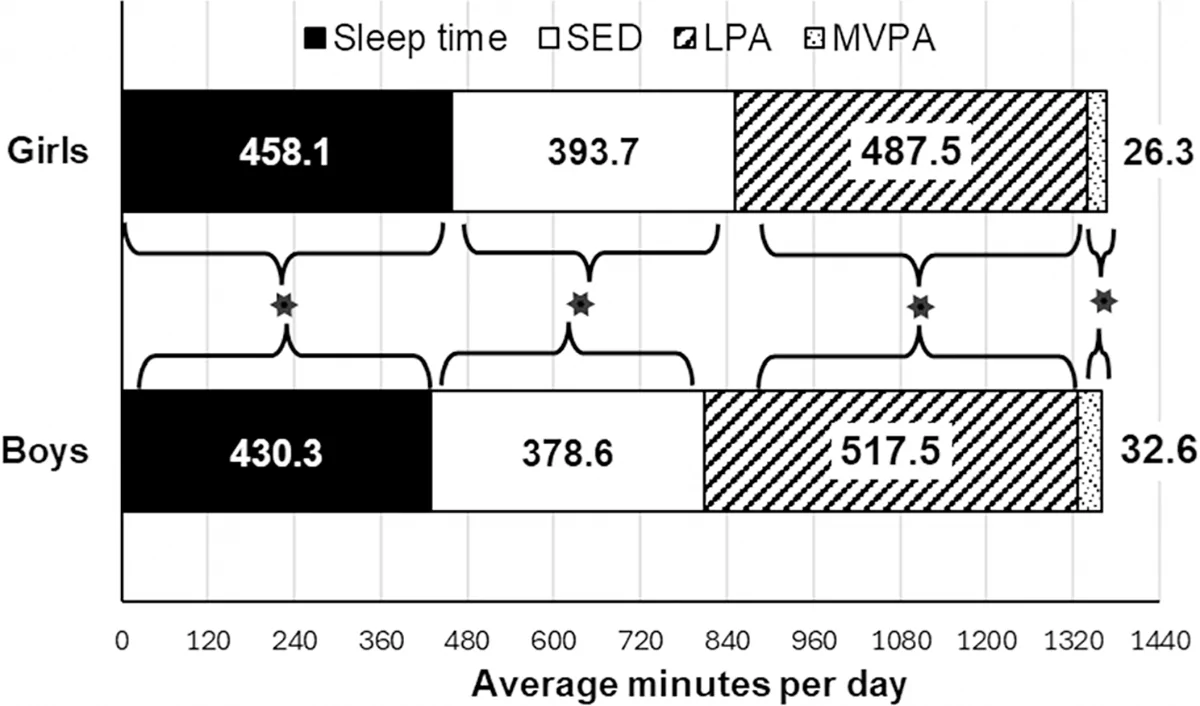
Average minutes of activity within the 24-hour activity cycle by sex. LPA = light physical activity, MVPA = moderate-to-vigorous physical activity, SED = sedentary behavior. Sleep time refers to 24-hour total sleep time across the day. Star and brackets indicate significantly different amount of time spent in given movement activity

Findings displayed in Fig. 1 show significant sex differences in 24-hour movement behaviors. Girls had significantly less time spent in LPA (girls [G]: 487.5±4.7 min/day vs. boys [B]: 517.5±5.3 min/day; *p*_raw_ and *p*_FDR_<0.001) and MVPA (G: 26.3±0.9 min/day vs. B: 32.6±1.2 min/day; *p*_raw_ and *p*_FDR_<0.001). Adolescent girls spent significantly more time in sedentary behavior than their male peers (G: 393.7±4.6 min/day vs. B: 378.6±5.6 min/day; *p*_raw_ and *p*_FDR_=0.037). Total sleep time was longer among girls than boys (G: 458.1±3.0 min/night vs. B: 430.3±2.9 min/night; *p*_raw_ and *p*_FDR_<0.001).

### Race/Ethnicity Differences in 24-Hour Movement Behavior

**Fig. 2:**
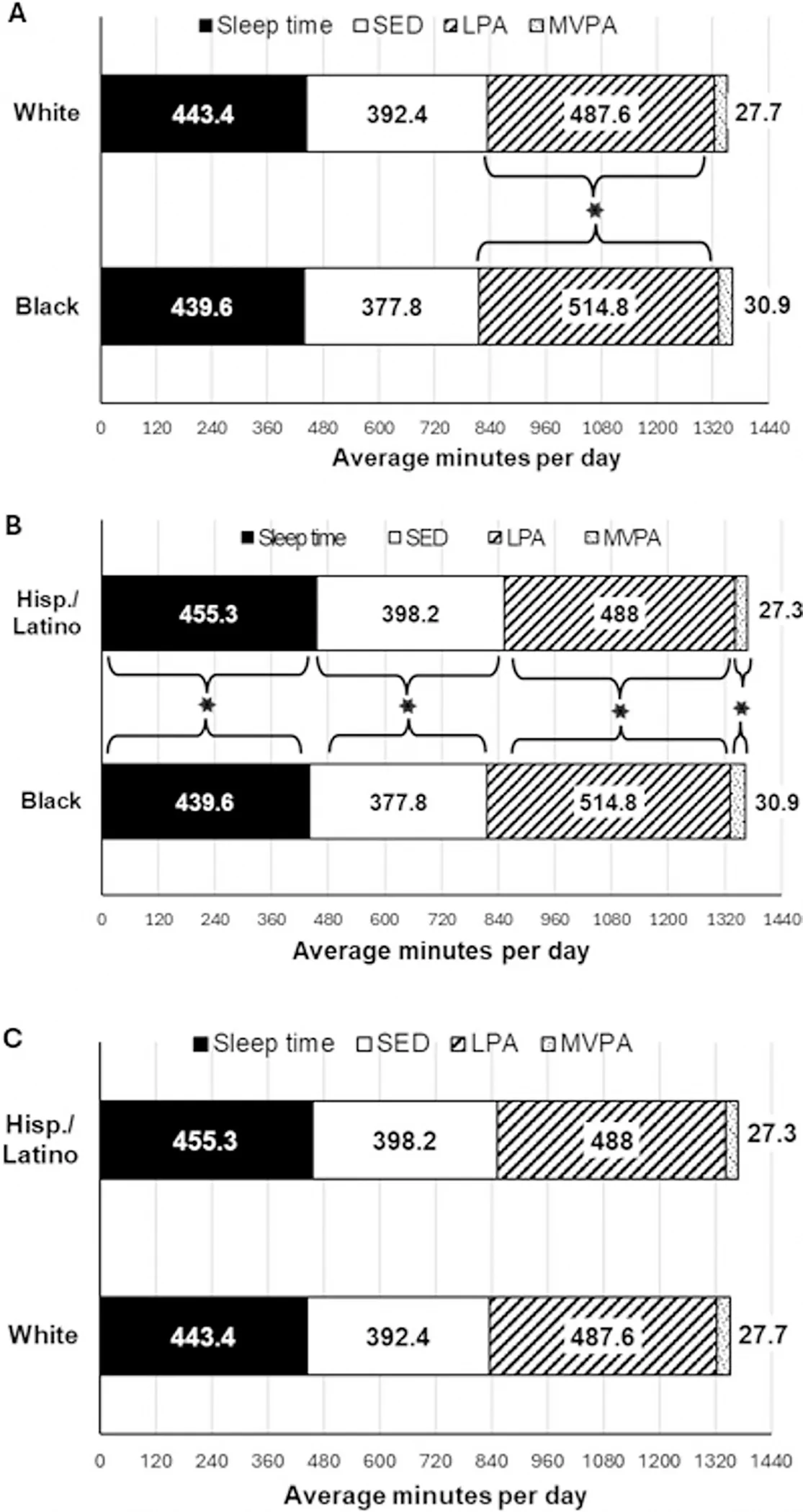
Average minutes of activity within the 24-hour activity cycle by race/ethnicity. LPA = light physical activity, MVPA = moderate-to-vigorous physical activity, SED = sedentary behavior. Sleep time refers to 24-hour total sleep time across the day. Star and brackets indicate significantly different amount of time spent in given movement activity. Panel **A** (comparison between Black and White adolescents); panel **B** (comparison between Black and Hispanic/Latino adolescents); panel **C** (comparison between Hispanic/Latino and White adolescents)

There were significant differences by race and ethnicity in daily total sleep time, sedentary time, and time spent in LPA and MVPA between groups (see Fig. 2). Non-Hispanic Black adolescents spent more time daily in LPA than non-Hispanic White adolescents (Black: 514.8±5.3 min/day vs. White: 487.6±7.7 min/day; *p*_raw_=0.006 and *p*_FDR_=0.023). There were no other differences in time spent in 24-hour movement behaviors (i.e., sedentary time, MVPA, and sleep time) between Black and White youth (all *p* >.05).

Among non-Hispanic Black and Hispanic/Latino adolescents, differences were observed across all 24-hour movement behaviors (see Fig. 2). Black adolescents spent more time in MVPA (Black: 30.9±1.1 min/day vs. Hispanic/Latino: 27.3±1.2 min/day; *p*_raw_ *and p*_FDR_=0.039) and LPA (Black: 514.8±5.3 min/day vs. Hispanic/Latino: 488.0±5.9 min/day; *p*_raw_=0.001 and *p*_FDR_=0.004). Hispanic/Latino adolescents had more sedentary time (Black: 377.8±5.2 min/day vs. Hispanic/Latino: 398.2±6.1 min/day; *p*_raw_=0.014 and *p*_FDR_=0.019) and longer daily total sleep time (Black: 439.6±3.0 min/day vs. Hispanic/Latino: 455.3±4.0 min/day; *p*_raw_=0.002 and *p*_FDR_=0.004) compared with non-Hispanic Black adolescents.

Lastly, when comparing non-Hispanic White and Hispanic/Latino participants, total sleep time was marginally higher among Hispanic/Latino adolescents compared with White adolescents (*p*_raw_=0.051 and *p*_FDR_=0.20) (Fig. 2). No other differences in 24-hour movement behavior components were observed between both groups (all *p*>.05).

### Proportion of Teens Across Subgroups Meeting Recommendations

Table 2 shows the proportion of adolescents meeting the aerobic physical activity recommendations of at least 60 min of MVPA/day and the minimum sleep recommendations of 8 h/day across sex and race/ethnicity groups. In total, 8.1% of the sample met the physical activity recommendations, and 23.4% met the minimum sleep recommendations. Only 4.3% of girls in the sample met the physical activity recommendations compared with 12.3% of the boys (*p*<.001), whereas 31.5% of girls in the sample met the minimum sleep recommendations compared with 14.4% of the boys (*p*<.001). Across race/ethnicity, Black adolescents had the highest percentage of meeting the physical activity recommendations at 10.2%, followed by White (6.6%) and Hispanic/Latino youth (5.3%). For sleep, Hispanic/Latino youth had the highest percentage of meeting the recommendations at 30.6%, followed by Black (20.7%) and White (19.9%) adolescents. There was a significant overall difference in those meeting the sleep recommendations by race (*p*=.016), but not for the physical activity recommendations (*p*>.05).

## Discussion

This study characterized 24-hour movement behaviors (MVPA, LPA, sedentary behavior, and sleep) among an ethnoracially and socioeconomically diverse sample of US adolescents, examining sex and racial/ethnic differences using objective measures. We found differences in the components of the 24-hour movement behaviors by sex and race/ethnicity. Some, but not all, of our hypotheses were supported. While disparities in physical activity engagement and sleep were postulated, Black adolescents surprisingly had the highest levels of physical activity overall and had higher levels of LPA than their White peers.

We found that adolescent girls engaged in significantly less MVPA and LPA but spent more time sleeping per day compared to boys. The physical activity findings are consistent with much of the physical activity surveillance literature among youth. There has been longstanding evidence that girls engage in less structured activity than boys during the adolescent period [33, 34]. Several hypotheses have been posited for the lower physical activity during adolescence for girls versus boys, including stereotype and gender norms related to body shape and size [35–37]. While girls within the sample had less time engaging in MVPA and LPA, conversely, girls spent nearly 30 more minutes sleeping than boys. Studies examining sex differences among adolescents in sleep measures such as sleep duration and sleep quality have been mixed, but have generally pointed to greater irregularity and longer sleep duration among adolescent girls [25, 38–40]. It has been posited that these sex differences in sleep patterns may be influenced in part by pubertal onset [41], with boys having a later onset of puberty in addition to other potential social and contextual factors [40].

Beyond sex differences in 24-hour movement behaviors, we also found evidence for racial differences. Specifically, Black adolescents had more time spent in LPA than their White and Hispanic/Latino peers, while also spending less time sedentary than their Hispanic/Latino peers. Other observations included significantly shorter total sleep time among Black adolescents compared with Hispanic/Latino adolescents. While it would have been encouraging to see high levels of MVPA across all race/ethnic groups, the higher engagement in LPA among Black youth is a reasonably positive finding, particularly when considering accumulating evidence regarding the cardiometabolic and learning/education-based (e.g., academic performance) benefits of LPA among children [42–44]. Infusing LPA into classrooms through activity breaks to interrupt sedentary behavior (i.e., “brain breaks”) may be a feasible way of countering childhood health disparities to improve health and educational outcomes among children. Programs such as the *Interrupting Prolonged Sitting with Activity (InPACT)* program have been shown to enhance movement and learning even in lower-resourced settings/schools [45, 46]. Additionally, LPA can also be an easier shift from being sedentary and may support progression to higher-intensity activity, as well as promote positive experiences that can foster sustained engagement of physical activity in later years [47]. With regard to sleep, the observation that Black adolescents had shorter total sleep time compared with Hispanic/Latino adolescents is in line with and supported by prior studies [7, 12, 25, 48]. For example, Yip and colleagues in their sample of adolescents ages 13–15 years, found that Black adolescents slept 36 min less than Latinx youth [48]. The shorter sleep duration experienced by Black adolescents may be due in part to contextual factors such as racial discrimination and neighborhood disadvantage [49, 50]. Thus, continued interventions are needed to counter racial discrimination and improve the neighborhood and built environment where youth live to improve adolescent health and well-being.

Among the sample participants, adherence to the recommended amount of time for each component of the 24-hour movement behaviors was low. More specifically, according to the US physical activity and sleep guidelines, adolescents need ≥ 60 min of MVPA and 8–10 h of sleep daily [19, 22]. We observed that approximately 8% of the sample met the US guidelines of 60 min of daily MVPA, while only 23.4% met the minimum 8 h of recommended sleep for US adolescents. On average, participants had 445 min (~ 7.4 h) of sleep time, 29 min of MVPA, and 387 min (~ 6.5 h) of sedentary time daily. The average minutes of daily total sleep time was 35 min less than the minimum of 8 h, whereas the 29 min of MVPA was less than half of the recommendation. While sedentary recreational screen time was not measured directly, the 387 min of sedentary time is equivalent to over 6 h of sitting. Contextualizing our findings with recent results from adolescent samples using large US datasets such as the Youth Risk Behavior Surveillance System (YRBSS) and the National Survey of Children’s Health, our sample had lower percentages of participants meeting the physical activity and sleep guidelines [23, 51]. Only about 8% and 23% of our sample met the movement and sleep guidelines, respectively, compared with 26.9% and 28.5% of the YRBSS study sample [23]. Researchers have also pointed to the increasing time spent on screen-based devices among children and adolescents, with recent data from the National Health Interview Survey for teens indicating that about half of US youth report four or more hours of daily screen time [52, 53]. Similarly, another study using the FFCWS cohort, reported that adolescents spend over 5 h on screen-based devices, communicating with friends on average for 2 h/day, watching TV and videos for nearly 2 h/day, and playing games on computers or handheld devices for over an hour/day [54]. In short, adolescents in our study and those nationally are obtaining insufficient sleep, engaging in prolonged sitting, and are not physically active. While evidence within the literature has pointed to American adolescents not meeting the different components of the 24-hour guidelines—MVPA, sleep, and sedentary behavior [10]—our study further corroborates how wide the gap is between the recommendations and actual objective measurement of these activity behaviors among a diverse sample of US adolescents.

The observations of this study should be interpreted considering a few limitations and caveats. First, this study is cross-sectional and only represents one point in time rather than reflecting trends in activity across the lifespan. Additionally, while sedentary behavior was measured across all groups, it was not possible to delineate between screen-based sedentary behavior and total sedentary behavior. Future studies should incorporate measures of device-based behaviors to help provide an understanding of the drivers of inactivity. Lastly, study participants were from a national birth cohort of participants recruited from 20 urban US cities. Thus, the generalizability of the study findings to adolescent groups outside of the US or to those born in rural communities is uncertain. Nevertheless, despite these limitations, our study had several strengths, including the comprehensive characterization of 24-hour movement behaviors among a large, diverse sample of adolescents using objective measures of movement. Future analyses of the FFCWS cohort should track whether the 24-hour movement behaviors and disparities by demographic groups have changed (e.g., improved, declined, remained stable) into adulthood. This may, in turn, inform longitudinal models of how adolescent behavioral habits evolve into adult outcomes, to better anticipate determinants of chronic disease in the future.

## Conclusion

In summary, we characterized the 24-hour activity cycle among a large, diverse, national sample of adolescents, providing insight into the patterns of activity among youth that will better inform future interventions targeted at improving health behaviors. Adolescents are not engaging in enough physical activity nor obtaining sufficient sleep across sex and race/ethnicity groups, and sex differences in physical activity and sleep adherence persist in this sample. Inadequate sleep and physical activity among youth are major health threats, increasing risk for future cardiovascular and metabolic disease [55–57]. Continued work is needed to characterize 24-hour movement behaviors among US adolescents, with the potential benefit of improved physical and mental health among U.S. youth. Given the consistent sex gap in youth physical activity engagement, renewed focus on equitable sex/gender-based interventions on sleep and physical activity is also needed.

## Figures and Tables

**Table 1:** Sample sociodemographic characteristics

Characteristic	Mean (SD) or %	(Min; Max)

Sociodemographic		
Age (years)	15.4 (0.6)	(15; 18)
Sex at birth (%female)	52.7%	--
Race/ethnicity (%)		--
*Non-Hispanic Black*	51.4%	
*Hispanic/Latino*	29.3%	
*Non-Hispanic White*	19.3%	
Pubertal development vs. peers (%)		--
*Earlier*	20.8%	
*About the same*	59.8%	
*Later*	19.4%	
BMI-for-age percentile (%)	72.1 (25.6)	(1; 99)
Household income, yearly (USD)	$60,247 ($58,310)	($0; $530,000)
Caregiver education (%)		--
*Less than high school*	17.1%	
*High school diploma/GED*	18.8%	
*Some college*	46.1%	
*Bachelor’s degree or greater*	18.0%	
24-hour movement behaviors (min/day)		
LPA	501.7 (94.6)	(75; 803.6)
MVPA	29.2 (19.7)	(0; 140.7)
Sedentary time	386.6 (95.2)	(123; 749.5)
Total sleep time	445.0 (57.1)	(250; 735)

*BMI* body mass index, *GED* general educational development, *LPA* light physical activity, *MVPA* moderate to vigorous physical activity, *USD* United States dollar

**Table 2:** Proportion of adolescents meeting aerobic physical activity and sleep recommendations

	MVPA recommendations		Sleep recommendations	
		
	# meeting rec/total *n*	%	# meeting rec/total *n*	%

Total Sample	57/704	8.1	165/704	23.4
Boys	41/333	12.3	48/333	14.4
Girls	16/371	4.3	117/371	31.5
Non-Hispanic Black	37/362	10.2	75/362	20.7
Hispanic	11/206	5.3	63/206	30.6
Non-Hispanic White	9/136	6.6	27/136	19.9

*MVPA* moderate-to-vigorous activity, *rec* recommendations. MVPA recommendations were defined as engaging in at least 60 minutes of MVPA/day, and sleep recommendations were defined as obtaining at least 8 hours sleep/day

## Data Availability

Data are available upon reasonable request from the study Principal Investigators (LH, AMC).

## Code Availability

Available upon reasonable request.
